# Influence of Depressive Symptoms on Disease Activity in Patients with Rheumatoid Arthritis: The Mediating Effect of Self-Efficacy

**DOI:** 10.3390/medicina62030449

**Published:** 2026-02-27

**Authors:** Wen-Wei Lin, Chieh-Tsung Yen, Hua-Lung Huang, Hanoch Livneh, Ming-Chi Lu, Wei-Jen Chen, Tzung-Yi Tsai

**Affiliations:** 1 Graduate Institute of Sports Science, National Taiwan Sport University, Taoyuan 333325, Taiwan; pit543@yahoo.com.tw; 2 Department of Neurology, Dalin Tzu Chi Hospital, Buddhist Tzu Chi Medical Foundation, Chiayi 62247, Taiwan; dm335164@tzuchi.com.tw; 3 Department of Rehabilitation, Dalin Tzu Chi Hospital, Buddhist Tzu Chi Medical Foundation, Chiayi 62247, Taiwan; dm968414@tzuchi.com.tw; 4 Rehabilitation Counseling Program, Portland State University, Portland, OR 97207-0751, USA; livnehh@pdx.edu; 5 School of Medicine, Tzu Chi University, Hualien 97004, Taiwan; dm252940@tzuchi.com.tw; 6 Division of Allergy, Immunology and Rheumatology, Dalin Tzu Chi Hospital, Buddhist Tzu Chi Medical Foundation, Chiayi 62247, Taiwan; 7 School of Post-Baccalaureate Chinese Medicine, Tzu Chi University, Hualien 97004, Taiwan; 8 Center of Sports Medicine, Dalin Tzu Chi Hospital, Buddhist Tzu Chi Medical Foundation, Chiayi 62247, Taiwan; 9 Department of Medical Research, Dalin Tzu Chi Hospital, Buddhist Tzu Chi Medical Foundation, Chiayi 62247, Taiwan; 10 Department of Environmental and Occupational Health, College of Medicine, National Cheng Kung University, Tainan 70428, Taiwan

**Keywords:** depressive symptoms, disease activity score 28-joint count, self-efficacy, mediation, rheumatoid arthritis

## Abstract

*Background and Objectives:* Rheumatoid arthritis (RA) is a lifelong autoimmune disease in which joint inflammation is caused by a dysfunctional immune system. Synchronous depressive symptoms have been identified as determinants of disease progression, especially disease activity as measured using the 28-joint Disease Activity Score (DAS28). Although growing evidence suggests the interplay between depression and DAS28 in RA, related findings remain inconsistent, implying that a pivotal underlying mechanism is overlooked. Hence, we aimed to examine whether self-efficacy, the belief in one’s ability to manage health, could mediate the association between depressive symptoms and DAS28. *Materials and Methods:* Between January and October 2023, we carried out a cross-sectional survey to recruit patients with RA from a target hospital in Taiwan. Participants completed demographic and disease-related questionnaires, the Chinese version of the Arthritis Self-Efficacy Scale, and the Taiwanese Depression Questionnaire. Mediation analysis was performed using the Hayes PROCESS macro function on SPSS. *Results:* In 259 recruited persons with RA, depressive symptoms were found to negatively associate with self-efficacy, and self-efficacy negatively related to DAS28. Mediation analysis demonstrated that depressive symptoms affected DAS28 indirectly through self-efficacy (B = 0.022; 95% confidence interval: 0.015–0.031), accounting for 32.8% of the total impacts. *Conclusions:* Our findings suggest that the association between depressive symptoms and DAS28 may be mediated by individual self-efficacy. Interventions beyond the relief of depressive symptoms and that enhance the concept of self-efficacy should be emphasized while managing patients with RA.

## 1. Introduction

Inflammatory diseases are characterized by aberrant chronic inflammation that can affect the entire body. Rheumatoid arthritis (RA) is a representative example. The global population affected by RA increased from 17.5 million in 2020 to approximately 31.7 million in 2050, representing a 181% increase [1,2]. RA primarily affects synovial joints and leads to progressive cartilage loss and bone erosion. Consequently, the growing number of affected individuals is imposing a substantial economic burden on healthcare systems. In the United States, the total societal cost of RA was estimated at nearly $40 billion, which would approach $70 billion after adjusting for the 2025 US-dollar value [3,4]. On an average, the per capita healthcare expenditure for individuals with RA is $20,919, nearly three times higher than for those without RA ($7197) [5].

Beyond the significant economic impact, RA itself may incur severe mental health issues, particularly depressive symptoms, owing to the influx of inflammatory mediators and monoaminergic dysregulation [6]. A meta-analysis of 72 studies showed that depression was the most frequent comorbid condition associated with RA, with incidence ranging from 14% to 48% [7]. Additionally, patients with RA and concurrent depressive symptoms bear an overload of healthcare expenditure and double the likelihood of premature mortality relative to those with RA only [8,9]. To disrupt this vicious cycle, disease management strategies have shifted from treatment-focused models to proactive remedies that emphasize early management of disease progression [10]. For patients with RA, the 28-joint Disease Activity Score (DAS28), which assesses swollen and tender joints, acute phase response, and general health, is a widely employed index to quantify disease progression and therapeutic responses. Recent studies have examined the association between depression and DAS28. A prospective study that focused on the impact of depressive symptoms on disease activity in 400 RA cohorts reported that those with concurrent extreme depressive level have high DAS28 compared with those without depressive symptoms at baseline (*p* < 0.001) [11]. Similarly, another cross-sectional study supported a strong relationship between depressive symptoms and DAS28, reporting that patients with RA and attendant depression tended to experience a higher disease activity [12]. However, contrary to these reports, some investigators failed to elucidate a link between depressive symptoms and DAS28 [13,14,15]. These inconsistencies may stem from differences in study design, definitions, and participant characteristics. However, this reveals that additional factors may possibly buffer or modify the pathway between depressive symptoms and disease activity. Therefore, a detailed understanding of the crosstalk between depressive symptoms and DAS28 is required, without which any targeted interventions may not yield the required response.

In chronic disease management, self-efficacy, the belief in one’s capacity to successfully manage illness-related challenges, acts as a critical determinant of health outcomes [16]. This concept originated from the narrative by Albert Bandura in 1977, which referred self-efficacy as the confidence in one’s ability to succeed in specific situations or achieve a desired goal [17]. Specifically, a robust relationship has been identified between self-efficacy and clinical outcomes in patients with rheumatic diseases. One study indicated that self-efficacy is positively associated with DAS28 in patients with RA [18], primarily because it can act as a catalyst to strengthen patients’ adherence to medication regimes, improving treatment outcomes [16]. In a recent network analysis, a higher level of self-efficacy was significantly correlated with mental well-being in adolescents [19]. One study showed that for each unit increase in self-efficacy, the likelihood of incurring depression episode decreased by 7% [20]. Specifically, interventions aimed at enhancing self-efficacy have shown benefits in alleviating depressive symptoms in patients with chronic diseases [21]. Despite these evidences, few studies have examined whether self-efficacy can mediate the relation between depressive symptoms and DAS28 in RA. Identifying the association between these variables and their respective roles is helpful in understanding potential mechanisms and framing more efficient strategies to prevent functional decline. With this aim, we conducted this study to explore whether the relationship between depressive symptoms and DAS28 is mediated by self-efficacy.

## 2. Methods

### 2.1. Design and Study Population

Between January and October 2023, a cross-sectional survey using purposive sampling was conducted in the outpatient rheumatology and immunology clinic at a teaching hospital in Taiwan. Eligible participants (a) met the 2010 American College of Rheumatology diagnostic criteria for RA [22], (b) were aged ≥ 20 years at the time of the survey, and (c) were cognitively able to communicate with the interviewer. Participants who were unable to complete the questionnaire or undergo physical examination were excluded. This study was approved by the Institutional Review Board of the participating hospital prior to its commencement (No. B11201014), and all procedures were performed in accordance with the principles of the Declaration of Helsinki. Sample size estimation was based on an α level of 0.05, power of 0.8, and an assumed event rate of 0.2 [7,23]. In accordance with these indicators, a minimum of 191 participants were required to detect meaningful effects.

### 2.2. Assessments of Primary Indicators

The primary indicators included DAS28, depressive symptoms, and self-efficacy. Data pertaining to DAS28 were obtained from individual medical records. The DAS28 has four components: total number of tender and swollen joints, erythrocyte sedimentation rate (ESR), and visual analog scale scores for patient’s global health assessment. A higher DAS28 is indicative of more active disease. Self-efficacy and depressive symptoms were assessed using the Arthritis Self-Efficacy Scale (ASES) and the Taiwanese Depression Questionnaire (TDQ), respectively.

In 1980, Lorig et al. introduced ASES to quantify individual self-efficacy, and the scale has been widely adopted to appraise self-efficacy levels in patients with rheumatic diseases so far [24,25]. Three dimensions are considered and aggregated to measure self-efficacy over the past month: pain (five items), physical function (nine items), and other symptoms (six items). Each item is scored from 10 (bad) to 100 (good), with higher scores representing a greater self-efficacy [24]. The ASES has been translated into Chinese by Tsai et al. using backward translation [26]. Based on factor analysis and concurrent validity testing, the Chinese version of ASES (CASES) was deemed to have acceptable psychometric properties when applied to individuals with rheumatic disorders [26]. Herein, we used the CASES to measure individual self-efficacy. Cronbach’s alpha for the current sample was 0.91.

The TDQ was administered to assess depressive symptoms in all eligible patients. Its brevity and cultural appropriateness for Asian populations were the primary reasons for usage. This scale comprises 18 items related to depressive symptoms. Each item is rated on a 4-point frequency scale ranging from 0 (“never”) to 3 (“almost every day”) over the past week [27]. Therefore, total scores range from 0 to 54, with higher scores indicating greater psychological distress. Using the Diagnostic and Statistical Manual of Mental Disorders, 4th edition, as the gold standard, the TDQ demonstrated acceptable concurrent validity, with an area under the receiver operating characteristic curve of up to 0.92 [28]. Cronbach’s alpha for the current participants was 0.95.

### 2.3. Covariates

Based on previous studies and clinical experience [29], the covariates mainly included demographic and disease characteristics. Demographic variables consisted of age (continuous variable), sex (female or male), education (junior college or below vs. above junior college), living arrangements (alone or not), employment status (employed or unemployed), current smoking (yes or no), and current exercise (yes or no), which were collected using a self-administered questionnaire. Exercise status was defined as engaging in exercise at least three times per week. Disease characteristics included the presence of metabolic disorders (diabetes mellitus, hypertension, heart disease, or stroke), body mass index (BMI), RA duration, and use of biological disease-modifying anti-rheumatic drugs (DMARDs) for more than 3 months after RA onset.

### 2.4. Statistical Processing

**Figure 1:**
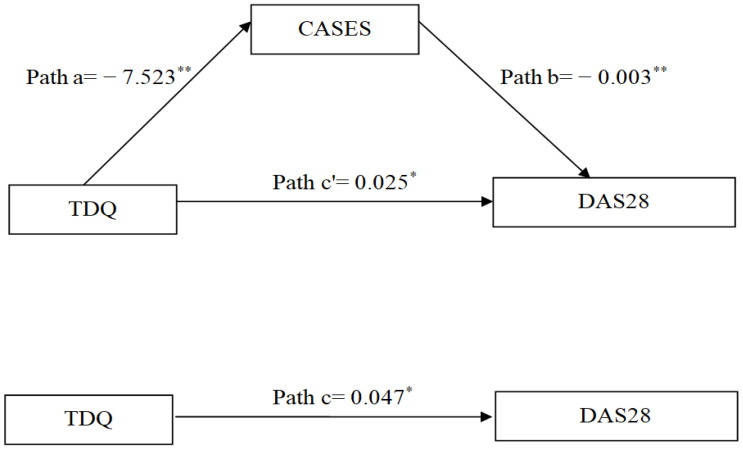
Diagram illustrating the mediating effect of self-efficacy on the relationship between depressive symptoms and DAS28. * *p* < 0.05; ** *p* < 0.01. TDQ, Taiwanese Depression Questionnaire; CASES, Chinese version of the Arthritis Self-Efficacy Scale; DAS28, 28-Joint Disease Activity Score.

Statistical analyses were performed using standard statistical software (SPSS 22.0; IBM Corp., Armonk, NY, USA). Descriptive statistics, including means, standard deviations (SD), frequencies, and percentages, were executed to summarize baseline characteristics, TDQ scores, DAS28, and CASES scores. Differences in DAS28 across sample characteristics were examined using Student’s *t*-test for continuous variables or the chi-square test for categorical variables, as appropriate. Pearson correlation coefficients were used to assess the relationships among TDQ, CASES and DAS28. The add-on Hayes PROCESS package for SPSS software was used to explore the mediating effect of self-efficacy between depression and DAS28 [30]. The outputs of the PROCESS macro in this model were as follows: (1) depressive symptoms and self-efficacy were significantly associated and shown in path a; (2) depressive symptoms and DAS28 were significantly associated and displayed in path c; (3) the bond between self-efficacy and DAS28 was significantly associated and shown in path b; and (4) depressive symptoms predicted DAS28 through individual self-efficacy and were depicted in path a*b. The null hypothesis assumed that mediation existed when the estimate of a*b was not equal to zero. The coefficient c’ represented the direct effect of depressive symptoms on DAS28 after excluding the influence from self-efficacy. All aforementioned indicators are presented in Figure 1. Mediation analysis was conducted using bias-corrected bootstrap methods with 5000 samples to generate 95% confidence intervals (CI). The assumptions of normality, linearity, and homoscedasticity were simultaneously affirmed. A significant mediating effect was confirmed when the 95% CI did not include zero.

## 3. Results

### 3.1. Participant Characteristics

Table 1 summarizes the demographic and disease characteristics of the participants. A total of 259 patients with RA were included, of whom 206 were women (70.2%). The mean age was 57.17 (SD, 12.32) years. Most participants were married (70.5%) and unemployed (69.1%). More than 80% reported cohabitation and were non-smokers. Nearly half had completed education below junior college (51.3%) and engaged in regular exercise (51.4%). Regarding disease characteristics, the mean RA duration was 11.31 ± 5.19 years, and approximately 60% of participants had metabolic disorders and took biological agents. The mean BMI was 26.40 kg/m^2^. Except for age and disease duration, most baseline characteristics were not significantly associated with DAS28 (Table 1).

### 3.2. Correlation Analysis

Pearson correlation coefficients for TDQ, CASES and DAS28 are presented in Table 2. Higher levels of depressive symptoms were negatively associated with self-efficacy (r = −0.42, *p* < 0.001) and positively correlated with DAS28 (r = 0.46, *p* < 0.001). Self-efficacy was also negatively associated with DAS28 (r = −0.44, *p* < 0.001). Collectively, these findings confirm the expected associations among the key study variables.

### 3.3. Mediation Effect

Before examining the role of self-efficacy in mediating the relation between depressive symptoms and DAS28, two covariates including age and RA duration were controlled for. The total effect of depressive symptoms on DAS28 was significant (path c: *B* = 0.047, *p* = 0.001), after adjusting for covariates. The direct effects of depressive symptoms on self-efficacy (path a: *B* = −7.523) and of self-efficacy on DAS28 (path b: *B* = −0.003) were also significant (*p* < 0.05). After adjusting for the mediator and covariates, the direct effect of depressive symptoms on DAS28 was attenuated but remained statistically significant (path c’: *B* = 0.025, *p* < 0.001). The indirect effect through self-efficacy was significant, as the 95% CI did not include zero (path a*b = 0.022, 95% CI = 0.015–0.031). Overall, the mediating effect of self-efficacy accounted for 32.8% of the total effect (Table 3 and Figure 1).

## 4. Discussion

Depression is a major comorbid psychological condition associated with RA [6], and accumulating studies have attempted to explore its impact, particularly on DAS28; however, the corresponding conclusions remain inconclusive [11,12,13,14,15]. A cause for concern in prior studies is the neglect of other potential buffer, particularly self-efficacy, which is a cornerstone in individuals afflicted with chronic diseases [31]. To the best of our knowledge, the present study is the first to integrate these three dimensions in the RA field, facilitating a comprehensive exploration of their interplay. Herein, we found that depressive symptoms negatively related to self-efficacy and that self-efficacy negatively associated with DAS28, both results consistent with priori works [18,19,20]. Importantly, the present study extends our knowledge that self-efficacy acts as a pronounced mediator of the correlation between depressive symptoms and disease activity.

After controlling for baseline demographic and clinical characteristics, this study revealed that depressive symptoms were strongly associated with elevated DAS28 through the mediating effects of self-efficacy, which accounted for 32.8% of the total effects. Although few head-to-head comparisons are available, the intermediating role of self-efficacy observed herein is consistent with earlier evidence manifesting that this belief serves as a crucial mediator in reducing serum cytokine concentrations [32,33]. Several mechanisms may explain this mediating pathway. First, the level of self-efficacy is positively associated with compliance with medication and health advice, even while facing challenges such as side effects or lifestyle changes. A recent survey conducted in 790 patients with hypertension reported a positive association between self-efficacy and medication literacy and that this belief could contribute to boosting medication adherence [34]. Former evidence converges to reveal that a stronger sense of self-efficacy and a greater perseverance and participation in shared decision-making facilitate access to health-related information and ultimately improves clinical outcomes [35,36]. Second, as reported previously [37], there is a positive correlation between higher self-efficacy and the inclination to use other complementary therapies, which in turn benefits symptom management to some extent. Taken together, our study attempts to clarify the correlation between depressive symptoms and DAS28 among patients with RA, and shows that management of DAS28 goes beyond the relief of psychological disturbances only; instead, it should be proactively driven by enhancing individual self-efficacy.

Regarding improvement in self-efficacy, several approaches may be employed to improve individual self-efficacy, including digital technologies and self-care management programs. Digital health technologies refer to the application of computing platforms, such as web-based dashboards, to streamline communication and learning through online delivery [38]. In a recent systematic review that focused on the correlation between self-efficacy and technology-based intervention, digital technologies substantially enhanced self-efficacy, together with the quality of health education received as compared to standard care [39]. Notably, some scholars presumed that patients with chronic disorders require increased support from healthcare providers despite engaging in e-learning approaches [40]. Thus, a hybrid model that can include digital tools in face-to-face interactions between physicians and patients might be more appropriate to enhance self-efficacy, particularly for older patients. Further, an embedded nurse-led case management (NLCM) program should be considered to combat this deepening crisis. In addition to streamlining healthcare access via the follow-up and interactive telecommunications, NLCM can improve the clinical prognosis of adults with RA by augmenting self-efficacy with a reduction in C-reactive protein (CRP) levels and inflammatory responses [41,42].

Despite the important public health implications of this study, some limitations and unresolved questions should be acknowledged. First, participants were exclusively recruited from a single hospital in Taiwan, which necessitates careful consideration while extending our findings to other ethnic populations. It would be advantageous to replicate similar studies across various local or regional institutions to enhance the generalizability of the results. Second, given the use of DAS28 with a focus on ESR, future researchers could employ an alternative indicator, such as DAS28-CRP, to determine whether the current findings can be replicated across different demographic and geographical groups. Third, the cross-sectional data applied provided information regarding the patient’s status at a single observation point, thus hardly clarifying the sequence among the variables included, as relevant exposure and mediating factors were measured at the same time. Thus, conducting a longitudinal study to validate the relationships reported here is critical and should be performed in the future. Finally, reliance on self-administered questionnaires to assess depressive symptoms may introduce misclassification. Nevertheless, prior evidence suggests that self-administered assessments do not overestimate depression in patients with chronic conditions [43]. Moreover, we used the TDQ to assess depressive symptoms because it is a cost-effective instrument for detecting major depression and recommending an appropriate intervention [27,28]. Notwithstanding these concerns, prior to embarking on this survey, we calculated the required sample size to ensure statistical power, and therefore, this study provides robust findings that self-efficacy mechanistically buffers the relation between physiological stress and changes in DAS28 and could serve as a reference in designing a more targeted care regimen.

## 5. Conclusions

The findings of this study can renew the current perspectives toward RA management, and highlight the role of self-efficacy in mediating association between depressive symptoms and subsequent disease activity. Based on the findings of mediation analysis, we found that self-efficacy did serve a critical mediating role in the influence of depressive symptoms on DAS28, accounting for a substantial proportion (32.8%) of the total effects. To our knowledge, this is the first exploration to evaluate this mediation effect in a large cohort of RA and highlight that enhancing self-efficacy can be an attainable non-pharmacological strategy to manage the disease activity in patients with RA. In clinical practice, it is crucial for practitioners and nursing staff in rheumatology clinics to recognize the significance of self-efficacy and to actively motivate patients with RA to participate in ongoing follow-up evaluations and reinforcement of this belief. Simultaneously, incorporating interdisciplinary collaboration to improve self-efficacy is indispensable in achieving optimal disease control in RA.

## Figures and Tables

**Table 1:** Differences in DAS28 by demographic and disease features recorded at baseline (n = 259).

Variables	Mean ± SD	n (%)	DAS28		
			Mean ± SD	t/r	*p*
**Demographic data**					
Age (years)	57.17 ± 12.32			−0.13 *	0.02
Married status					
Single		53 (20.5)	3.74 ± 1.44	t = −1.35	0.18
Married		206 (79.5)	3.44 ± 1.40		
Sex					
Male		54 (20.8)	3.12 ± 1.54	t = −0.33	0.14
Female		205 (70.2)	3.20 ± 1.37		
Educational level					
Junior college below		132 (51.0)	3.49 ± 1.44	t = −0.18	0.82
Junior college above		127 (49.0)	3.51 ± 1.33		
Job					
Unemployed		179 (69.1)	3.43 ± 1.31	t = −1.15	0.10
Employed		80 (30.9)	3.65 ± 1.61		
Living status					
Living alone		30 (11.6)	3.71 ± 1.42	t = −0.88	0.38
Cohabitating		229 (88.4)	3.48 ± 1.41		
Cigarette smoking					
Yes		33 (12.7)	3.52 ± 1.39	t = 0.56	0.57
No		226 (87.3)	3.37 ± 1.61		
Regular exercise					
Yes		133 (51.4)	3.36 ± 1.42	t = 1.58	0.12
No		126 (48.6)	3.66 ± 1.40		
**Disease characteristics**					
Disease duration (years)	11.31 ± 5.19			−0.24 **	0.001
Metabolic disorders					
Yes		153 (59.1)	3.62 ± 1.42	t = −1.58	0.11
No		106 (40.9)	3.34 ± 1.40		
Biological agents					
Yes		153 (59.1)	3.49 ± 1.36	t = 0.12	0.90
No		106 (40.9)	3.52 ± 1.48		
BMI	26.40 ± 4.26			0.49	0.60

SD, standard deviation; BMI, body mass index; DAS28: 28-Joint Disease Activity Score. * *p* < 0.05; ** *p* < 0.01.

**Table 2:** Relationships among the TDQ, CASES, and DAS28 (n = 259).

Variables	Mean	SD	TDQ	CASES	DAS28
TDQ	15.66	11.12	1	-	-
CASES	1613.04	318.74	−0.44 **	1	-
DAS28	3.51	1.14	0.42 **	−0.43 **	1

** *p* < 0.01.

**Table 3:** PROCESS model summary of mediation analysis.

Variables	Path c		Path a		Path c’ and b		Path a*b	
	β coef	SE	β coef	SE	β coef	SE	β coef	95% CI
TDQ	0.047	0.078 **	−7.523	1.781 **	0.025	0.008 *		
CASES	---	---	---	---	−0.003	0.003 **	0.022	0.015–0.031
Age	−0.005	0.067	−2.161	1.542	−0.007	0.001		
Disease duration	−0.021	0.015	5.378	3.493 *	−0.005	0.015		

β coef, β coefficient; SE, standard error; 95% CI, 95% confidence interval; TDQ, Taiwanese Depression Questionnaire; CASES, Chinese version of the Arthritis Self-Efficacy Scale. * *p* < 0.05; ** *p* < 0.01.

## Data Availability

The analytical data used herein are available from the corresponding authors on reasonable request.
